# Covid-19 Pneumonia and Ventilation-induced Lung Injury: A Case Report

**DOI:** 10.2478/rjaic-2020-0020

**Published:** 2020-12-31

**Authors:** Lieke H.A. van Gastel, Evelien A.N. Oostdijk, Stefanie Slot, Dolf Weller

**Affiliations:** 1 Intensive Care department, Maasstad Ziekenhuis, Rotterdam, The Netherlands

**Keywords:** COVID-19 pneumonia, ICU, mechanical ventilation, pneumomediastinum, barotrauma

## Abstract

We present the case of a 67-year-old male patient, who was admitted to the intensive care unit for hypoxemic respiratory failure due to severe COVID-19 pneumonitis, requiring mechanical ventilation. Despite close monitoring using transpulmonary pressure measurements and interventions to pursue lung-protective ventilation, the patient developed extensive barotrauma including a right-sided pneumothorax, subcutaneous emphysema and pneumomediastinum while on pressure support ventilation. We hypothesize that the high respiratory drive that COVID-19 patients seem to exhibit, combined with diffuse alveolar injury and increased alveolar pressure, resulted in gross barotrauma.

**Conclusion:**

The respiratory characteristics that COVID-19 patients seem to exhibit might expose those on mechanical ventilation to an increased risk of developing ventilation-induced lung injury. This case emphasizes that caution should be taken in the respiratory treatment of patients with COVID-19 pneumonitis.

## Introduction

Data on mechanical ventilation in patients with COVID-19 pneumonia from the current pandemic are limited. A substantial number of patients with COVID-19 pneumonitis meet the Berlin definition of acute respiratory distress syndrome (ARDS) on admission to the intensive care unit (ICU), but over 50% seem to exhibit characteristics rarely seen in ARDS. Identification of two phenotypes (ARDS-like and pneumonitis-like phenotypes) was proposed to provide some guidance in respiratory treatment [1, 2]. Barotrauma was previously reported in up to 2% of patients with severe COVID-19 pneumonitis [3]. To the best of our knowledge, this is the first COVID-19 case with reported transpulmonary pressure measurements before detection of gross barotrauma while on pressure support ventilation. We discuss potential (patho) physiological mechanisms and COVID-19 characteristics that may have played a role in the development and extent of barotrauma.

## Case presentation

This case report presents the case of a 67-year-old male patient with a past medical history of hypertension, who was admitted to the ICU with hypoxemic respiratory failure. He had been admitted to the hospital 2 days prior, with complaints of dyspnea and fever since 2 weeks. Laboratory tests showed elevated C-reactive protein of 98 mg/ml (normal range 0–10 mg/ml), normal leukocyte count of 6.5 × 10^9^/L (normal range 4.0–10.0 × 10^9^/L) and a decreased lymphocyte count of 0.7 × 10^9^/L (normal range 1.0–3.5 × 10^9^/L). Nasopharyngeal swab polymerase chain reaction for SARS-CoV-2 was positive. He was tested negative for influenza A and B viruses, respiratory syncytial virus, Legionella antigen and Streptococcus pneumoniae antigen. Chest radiography showed bilateral diffuse opacities. He was treated with cefotaxime.

On ICU admission, he was intubated and deep sedation was pursued. Lung-protective ventilation strategy was used. Neuromuscular blockade was administered by continuous infusion, and intermittent prone ventilation was applied because of persistent hypoxemic failure (PaO2/FiO2 ratio: 118).

A CT scan performed 9 days after ICU admission showed widespread ground-glass opacities along with bilateral pulmonary embolisms, for which anticoagulation therapy with dalteparin 10000 IE twice a day was started. Ten days after ICU admission, 1 gram of methylprednisolone was given for 3 days because of persistent respiratory failure. This was combined with cefotaxime, ciprofloxacin, anidulafungin and voriconazole empirically since his respiratory status was too fragile to perform bronchoalveolar lavage.

**Figure 1:**
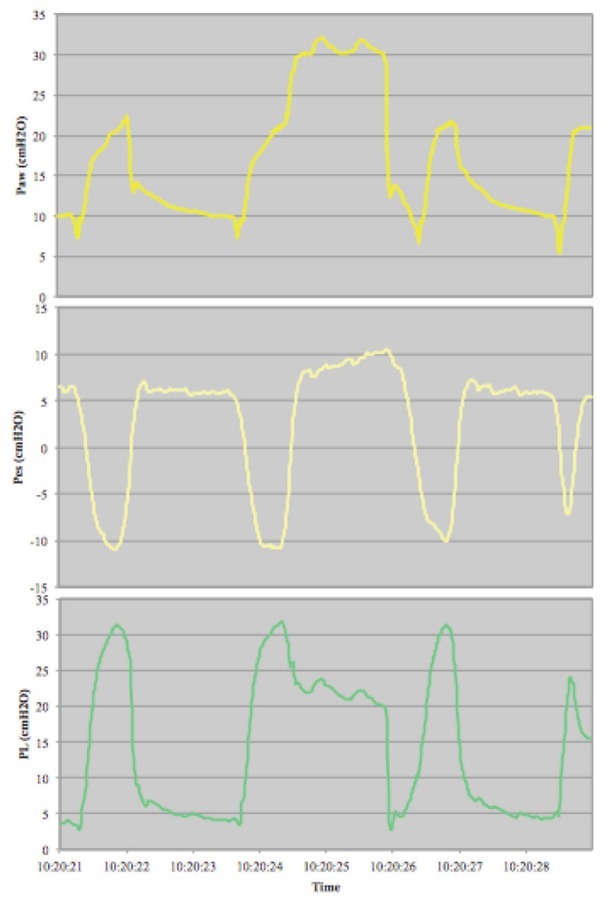
Transpulmonary pressure measurement during short inspiratory occlusion

After 4 weeks, sedation was slowly weaned to facilitate transition from pressure controlled to pressure support ventilation. This resulted in a high respiratory drive with large tidal volumes (9–12 ml/kg) and P0.1 values between 6.5–8.5 cmH_2_O while on pressure support ventilation with 10 cmH_2_O positive end-expiratory pressure and 10–14 cmH_2_O pressure support. A transpulmonary pressure measurement was done as shown in Figure 1. Measurements were performed using a Cooper Surgical esophageal catheter (connected to a Servo U mechanical ventilator by Getinge) during pressure support ventilation with short inspiratory occlusion. A normal dynamic compliance of 86 ml/cmH_2_0 was found. Airway pressure during inspiratory occlusion, i.e. plateau pressure, was 30 cmH_2_O. Substantial negative esophageal pressures, i.e. pleural pressures, and high transpulmonary pressures were observed as shown in Figure 1. The patient did not experience any pain, confusion or fear. Sedation was increased to lower respiratory drive (P0.1) and pressure support was gradually decreased to reduce tidal volumes in order to perform lung-protective ventilation.

**Figure 2:**
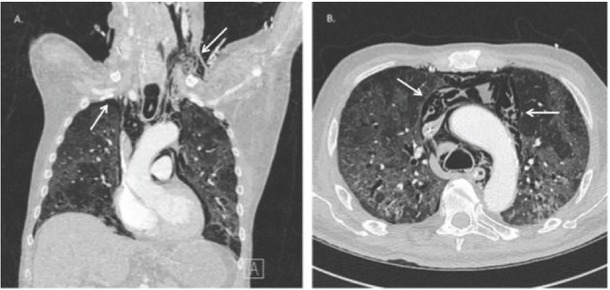
Chest CT showing (A) a right-sided apical pneumothorax and subcutaneous emphysema up to the left side of the neck and (B) extensive pneumomediastinum * Paw: airway pressure (cmH_2_O), Pes: esophageal pressure (cmH_2_O), PL: transpulmonary pressure (cmH_2_O)

In the absence of respiratory improvement, imaging was performed 2 days later. Chest radiography showed a right-sided apical pneumothorax, which was not yet present on X-ray 3 days prior. Subsequently, a chest CT scan was performed, as shown in Figure 2, confirming the presence of a right-sided ventral pneumothorax, subcutaneous emphysema up to the left side of the neck and an extensive pneumomediastinum. No airway injury was identified on CT; therefore barotrauma was considered to be the most likely cause. Conservative treatment with addition of antibiotics was chosen. The patient’s respiratory status gradually improved. He received a tracheostomy and was successfully weaned off the ventilator.

To the best of our knowledge, this is the first COVID-19 case with reported transpulmonary pressure measurements before detection of gross barotrauma during pressure support ventilation. The risk of barotrauma and volutrauma on controlled mechanical ventilation is well established, especially in ARDS. More recently, studies have elucidated that vigorous spontaneous patient effort can also potentially worsen lung injury, termed ‘patient self-inflicted lung injury’ (P-SILI). The same principles of barotrauma and volutrauma as well as stress and strain apply in mechanically ventilated, spontaneously breathing patients. Adopting the term ‘ventilation-induced lung injury’ has hence been suggested by some experts [4]. Prevention of P-SILI can be challenging since spontaneous effort has been shown to be difficult to keep within a safe range in the case of severe lung injury. Herein, it is presumed that the high respiratory drive overwhelms lung-protective reflexes, such as the Hering–Breuer inflation-inhibition reflex. Excessive negative pleural pressures generated by diaphragm contraction combined with large tidal volumes cause repeated overdistention of alveoli. Additionally, it is assumed that increased negative inspiratory pressures increase transmural vascular pressure and can thereby induce increased pulmonary perfusion and cause pulmonary edema. Importantly, distribution of pulmonary stress and strain is inhomogeneous, mostly affecting the dependent regions of the lung [5, 6].

Data on mechanical ventilation in COVID-19 patients are still limited. Although a substantial number of patients with COVID-19 pneumonia who are admitted to the ICU meet the Berlin definition of ARDS [1, 2], over 50% of patients exhibit characteristics that are rarely seen in ARDS such as near-normal respiratory system compliance. Gattonini et al. proposed identification of two phenotypes in COVID-19 patients: the ‘ARDS-like’ phenotype H and the ‘COVID-19 pneumonitis’ phenotype L, based on compliance, ventilation/ perfusion ratio, lung weight (on CT) and recruitability [2]. It seems plausible that the respiratory treatment offered should be adjusted to the (pheno)type of lung injury. Our patient exhibited respiratory characteristics of both phenotypes, namely bilateral pulmonary consolidations but with a normal compliance. This makes the extent of barotrauma in this case, which occurred despite close monitoring and interventions to pursue lung-protective ventilation, remarkable.

The high respiratory drive that patients with COVID-19 pneumonitis seem to exhibit increases stress and strain on the lungs as previously discussed, putting them at risk for P-SILI or ventilation-induced lung injury [2]. This might be aggravated by diffuse alveolar injury and increased alveolar pressure, as seen in severe COVID-19 pneumonitis, making the alveoli more prone to rupturing. Indeed, spontaneous pneumothoraces were seen in up to 2% of patients with severe COVID-19 pneumonia and a few cases even reported spontaneous pneumomediastinum [3, 7, 8, 9]. Furthermore, barotrauma was reported in 2% of mechanically ventilated patients with COVID-19 in the ICU [3]. One might therefore hypothesize that patients with COVID-19 pneumonitis are at increased risk of developing P-SILI or ventilation-induced lung injury, even in the pneumonitis-like phenotype, albeit a premature assumption. More large-scale research is warranted to confirm this.

## Conclusion

Mechanical ventilation in patients with COVID-19 pneumonitis in the current pandemic imposes a major challenge due to little available data and relatively unknown respiratory characteristics. COVID-19 patients seem to exhibit different phenotypes, a high respiratory drive, diffuse alveolar injury and increased alveolar pressure. This case emphasizes that caution should be taken in the management of respiratory failure in patients with COVID-19 on mechanical ventilation. Close monitoring of respiratory characteristics, using for instance transpulmonary measurement and pursuing lung-protective ventilation, also during pressure support modes, in order to prevent lung injury is crucial.
